# Intestinal obstruction in a patient with congenital transverse mesocolic defect with internal hernia: A case report

**DOI:** 10.1016/j.ijscr.2025.111121

**Published:** 2025-03-04

**Authors:** Samrat Shrestha, Suresh Maharjan, Bijay Raj Bhatta, Mecklina Shrestha, Sabin K. Ghimire

**Affiliations:** a National Academy of Medical Sciences, NAMS, Bir Hospital, Department of General Surgery, Kathmandu, Province-3, Nepal; b College of Medical Sciences(CoMS), Department of Emergency Medicine, Kathmandu, Province-3, Nepal

**Keywords:** Internal hernia, Transverse mesocolic hernia, Laparotomy, Intestinal obstruction, Congenital, Case report

## Abstract

**Introduction and importance:**

Internal hernias (IH) account for 5.8 % of all cases of intestinal obstruction. Intestinal obstruction due to congenital transverse mesocolic defects is a rare but significant clinical challenge that requires prompt recognition and surgical intervention.

**Case discussion:**

A 71-year-old female with COPD presented with acute abdominal pain, abdominal distension, and vomiting. On physical examination, the abdomen was distended with generalized tenderness. Contrast-enhanced computed tomography (CECT) revealed a transition point at the ileum. Failure of conservative management led to exploratory laparotomy that revealed a 7 cm ∗ 6 cm defect in the transverse mesocolon with herniation of ileal loops into the lesser sac. The hernia was reduced, and the defect closed. Postoperatively, the patient recovered well without complications.

**Clinical discussion:**

IH, including transverse mesocolic IH (TMIH), are often asymptomatic or present with vague abdominal pain. They are challenging to diagnose clinically and radiologically. It has been reported in several studies, with internal hernias due to mesocolic defects accounting for about 0.2–0.9 % of all abdominal hernias. CECT offers a diagnostic accuracy of 77 %. Surgical intervention is necessary for complicated cases, with a focus on reducing the herniated bowel and closing the defect. This case highlights the importance of considering congenital mesocolic defects in the differential diagnosis of unexplained intestinal obstruction, particularly in elderly patients.

**Conclusion:**

Congenital TMIH should be considered in the differential diagnosis of patients with unexplained bowel obstruction, especially when the clinical picture is atypical. Early diagnosis with imaging and surgical repair leads to favorable outcomes, as demonstrated in this case.

## Highlights

• Internal hernias (IH), especially from congenital transverse mesocolic defects, are rare (5.8% of all intestinal obstructions).
• CECT of the abdomen is the imaging modality of choice for diagnosing internal hernias, with an accuracy of 77%.
• Surgery is the primary treatment for complicated IH, involving the reduction of herniated bowel and closure of the defect.
• Timely surgical intervention is crucial to prevent bowel ischemia and reduce morbidity and mortality.
• Although rare, congenital transverse mesocolic IH should be considered in elderly with unexplained intestinal obstruction.

## 1 Introduction

Internal hernia (IH) is a rare entity occurring through a mesenteric/mesocolic defect that confines within the abdominal cavity. Acquired mesocolic internal hernias are common after abdominal surgery; however, congenital transverse mesocolic internal hernias are extremely rare. IH accounts for 5.8 % of all obstruction cases. Due to its rarity, IH is a diagnostic dilemma [1]. Contrast-enhanced computed tomography (CECT) with an accuracy of 77 % is an imaging modality of choice. After prompt diagnosis, timely surgical intervention is the mainstay of treatment to preserve the viability of the bowel and prevent resection of bowel segments, reducing morbidity and mortality [2]. We report a case of a 71-year-old female who presented with the feature of intestinal obstruction and underwent exploratory laparotomy to reveal a transverse mesocolic internal hernia. This case has been reported according to the revised SCARE guidelines 2023 [3].

## 2 Case presentation

**Fig. 1:**
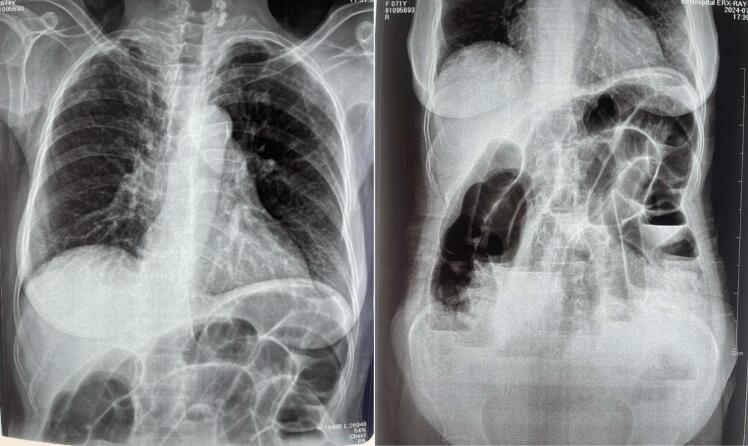
X-ray chest and abdomen erect view showing dilated bowel loop with multiple air-fluid levels suggesting intestinal obstruction.

**Fig. 2:**
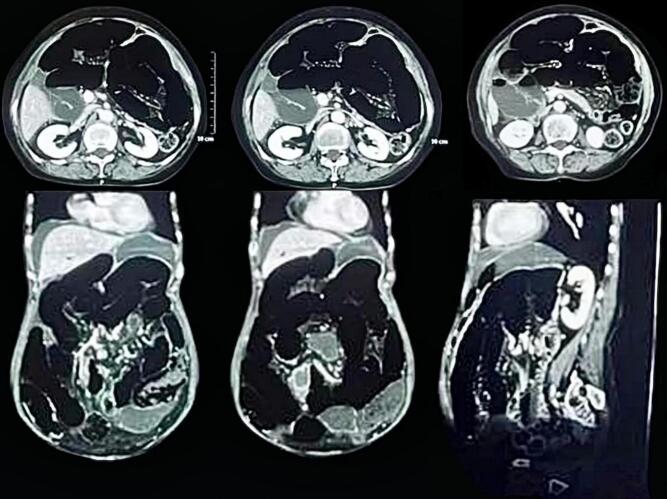
CECT axial, coronal, and sagittal views show dilated small bowel loops suggesting intestinal obstruction.

**Fig. 3:**
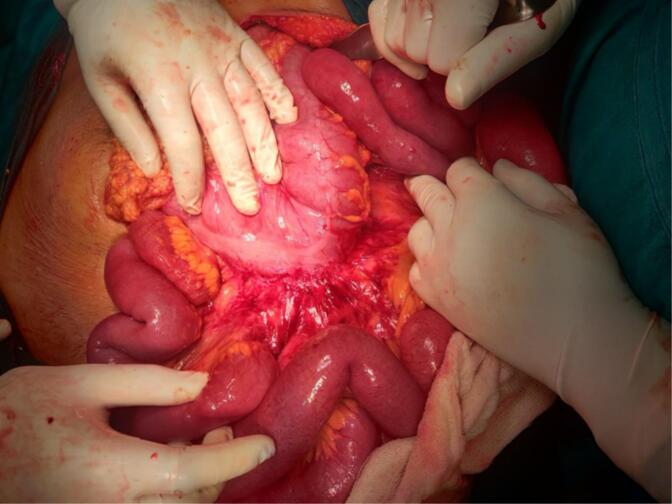
Intraoperative image showing adhesion of distal ileum and its mesentery with transverse colon.

**Fig. 4:**
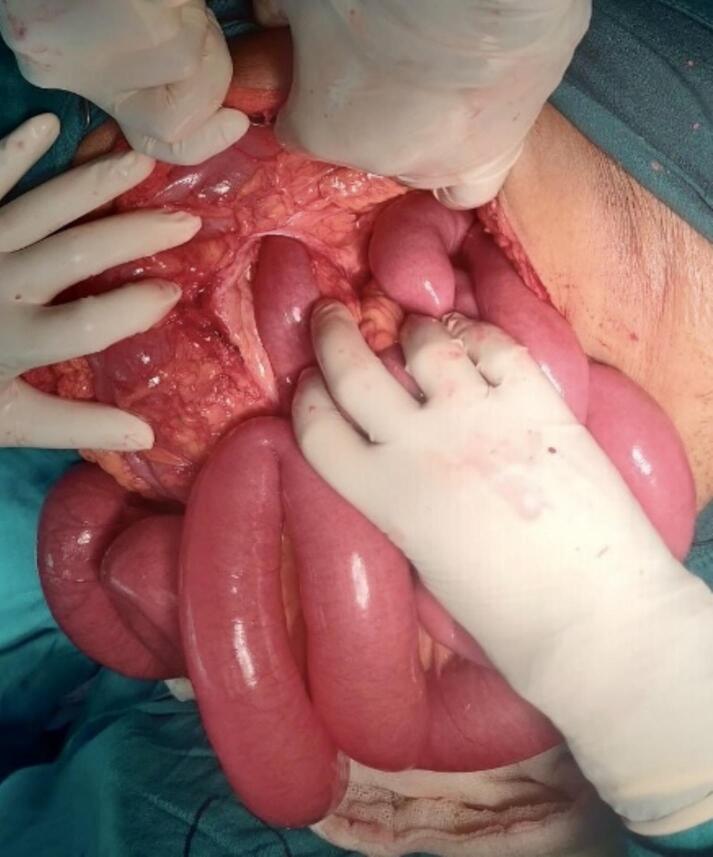
Intraoperative photograph showing herniation of healthy ileal loop through the transverse mesocolic defect demonstrated after the release of adhesion between the ileum and transverse colon.

**Fig. 5:**
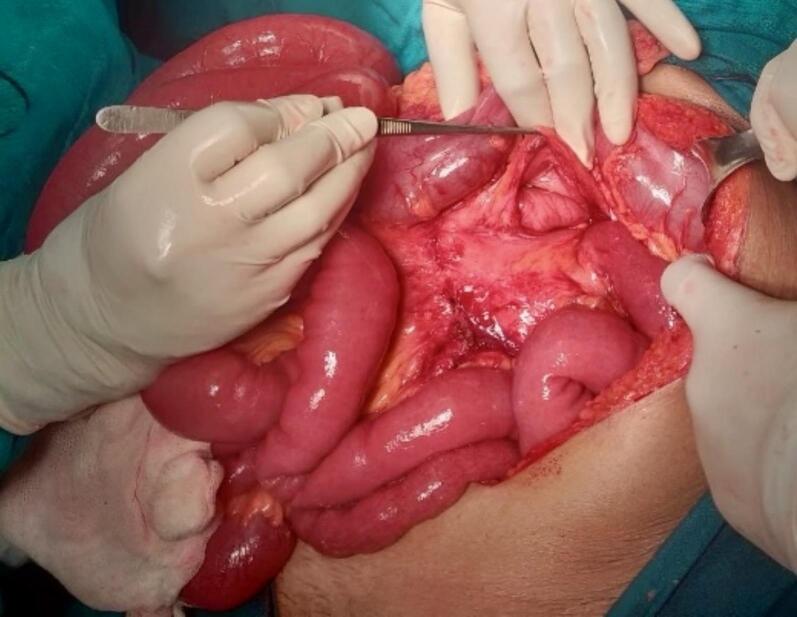
Intraoperative photograph showing defect at transverse mesocolon after reducing the herniated ileum.

A 71-year-old female patient with COPD presented to the emergency department of our hospital with the complaint of acute onset generalized abdominal pain with abdominal distention that started 5 days prior, which was associated with multiple episodes of vomiting (initially nonbilious later bilious). The patient had not passed flatus for 3 days. The patient had no significant history of abdominal trauma or previous abdominal surgeries. The patient had no other significant medical history. The patient was tachycardic with a pulse rate of 110 beats/min, and her blood pressure was 110/60 mmHg. The physical examination revealed a distended abdomen with generalized tenderness over all quadrants with hyperactive bowel sounds. Hernial orifices were intact. Total leukocyte count was 11,500/cumm with neutrophils of 88 % and lymphocytes of 10 %. Rest all the laboratory parameters were within normal limits. The patient's plain erect and supine abdominal X-ray showed dilated small bowel loops with multiple air-fluid levels suggesting small bowel obstruction (Fig. 1). CECT abdomen was done, which revealed a transition point at the mid part of the ileum with upstream dilatation of small bowel loops (maximum up to 4 cm) with congestion of mesenteric vein near the transition point (Fig. 2). The patient was resuscitated and admitted with a nasogastric tube, kept nil per oral (NPO), and maintenance intravenous fluid was added. Given the patient's advanced age and underlying COPD, careful consideration was given to fluid resuscitation. The patient received maintenance intravenous fluids with close monitoring of cardiac and pulmonary status. Fluid volumes were adjusted to avoid exacerbating potential pulmonary edema or heart failure, common concerns in elderly patients with compromised cardiovascular reserves. The patient's fluid balance was monitored regularly, and fluid therapy was titrated according to vital signs, renal function, and clinical response. The patient was admitted for conservative management with close monitoring. On 1st and 2nd day of admission, pain was persistent, and the patient did not pass flatus. After the 3rd day of admission, the patient had worsening abdominal pain, and abdominal distention was not relieved. The nasogastric tube drain contained 300–400 ml of bilious collection daily up to the 3rd day of admission. Exploratory laparotomy was planned as the patient showed no sign of improvement on conservative management. Intraoperatively, approximately 200 ml of serous ascitic fluid was drained from the peritoneal cavity. Adhesion between the ileum and its mesentery with the transverse colon was noted (Fig. 3). After releasing the adhesion, a 7 cm ∗ 6 cm defect in the transverse mesocolon was observed with herniation of healthy ileal loops into the lesser sac (Figs. 4, 5). The herniated ileal segment was reduced, and the mesenteric defect was closed with polyglactin 910, 3-0 suture in an interrupted fashion. The entire small bowel was inspected thoroughly, and no evidence of ischemia was noted. Postoperatively the patient was kept in the ICU for further monitoring. On the 2nd postoperative day (POD), the patient passed flatus, oral feeding with a liquid diet was started, and a normal diet was started on the 5th POD. The patient was subsequently discharged on the 7th POD. In 2-week, 1-month, and 6-month follow-ups, the patient did not have any symptoms of intestinal obstruction and recovered completely. The timeline of events from symptom progression, interventions, and follow-up is depicted in Table 1.

**Table 1:** Timeline of events from symptom progression, interventions, and follow-up.

Day	Symptom/Observation	Intervention/Action
Day 0	Acute onset of generalized abdominal pain, distention, and vomiting (initially nonbilious, later bilious)	Presented to ED; abdominal X-ray showing small bowel obstruction
Day 1	Abdominal pain persists; no flatus for 3 days	Nasogastric (NG) tube insertion, NPO, and IV fluids started
Day 3	Worsening abdominal pain and distention, bilious NG tube output (300–400 ml/day)	CECT abdomen shows transition point at ileum, exploratory laparotomy planned
Day 4	Surgical intervention (exploratory laparotomy)	Release of adhesions, reduction of hernia, closure of the mesocolic defect
Day 5	Postoperative recovery; passed flatus	Initiated liquid diet
Day 7	Postoperative recovery	Discharged from hospital
Week 2	Follow-up visit	No symptoms of obstruction
Month 1	Follow-up visit	No symptoms of obstruction
Month 6	Follow-up visit	Full recovery, no symptoms

## 3 Discussion

Internal hernias (IHs), the protrusion of the viscera through the peritoneum or mesentery but remaining within the abdominal cavity, are a rare entity with an incidence of less than 1 % [1]. Intestinal obstruction is the most common presentation in various IHs and accounts for up to 5.8 % of intestinal obstruction with a mortality rate as high as 50 % [4]. This condition can be broadly classified as congenital or acquired. Congenital IHs, either through normal foramen or through abnormal anatomical defects are thought to be due to intestinal malrotation in embryonic periods. Congenital IHs in adults are even rarer. IHs in adults are mostly acquired conditions, due to previous intra-abdominal surgery or sometimes because of trauma. Previous studies have primarily focused on acquired IHs, particularly those resulting from surgical procedures such as gastrojejunostomies and Roux-en-Y operations, whereas congenital transverse mesocolic internal hernias (TMIHs) remain extraordinarily rare [[5], [6], [7]]. Only a few cases of TMIH have been documented in the literature, with the majority of these involving patients who were younger, often with no significant co-morbidities [8]. Our case contrasts with many of these reported cases by presenting an elderly female (71 years) with underlying COPD, which may complicate management due to the patient's age and coexisting respiratory condition.

Congenital IHs, as described by Meyers, are mainly seven types based on their anatomical locations. The most common type is para duodenal, which accounts for 54 % of all cases, followed by pericecal (13 %), foramen of Winslow (8 %), transmesenteric or transmesocolic (8 %), supravesical and pelvic (6 %), and lastly transomental (4 %) [6]. Transmesenteric IH are divided into three main subtypes: small bowel mesenteric defect, transmesocolic, and Peterson's hernia [7]. Various hypotheses have been postulated for the cause of anatomical defects in the mesocolon or mesentery. Federschmidt proposed that the defects are due to partial regression of the dorsal mesentery, while Menegaux stated that fenestration occurred during the enlargement of inadequately vascularized areas. But commonly believed postulation is a defect due to developmental ischemic injury to the mesentery. The concurrence of intestinal atresia along with congenital mesenteric defects supports this hypothesis [8]. IHs can be asymptomatic if easily reducible or sometimes cause vague abdominal pain, colicky periumbilical pain, dyspepsia, or rare features of intestinal obstruction. On abdominal examination, a tender mass representing the Gordian knot of the herniated bowel loops can be felt in a few of the cases, distended abdomen in obstructed cases, and features of peritonitis in strangulated hernias [9]. Due to its wide range of presenting symptoms, clinical diagnosis of TMIH is extremely difficult, and there will be only modest help from radiological investigations. CT scan of the abdomen may show crowding of bowel loops in one location with the absence of bowels in their normal location, with the occasional ‘whirlpool sign’ of volvulus [10]. The ‘bag of bowels’ in the hernial sac with other findings makes the CT abdomen a sensitive tool with a diagnostic accuracy of 77 % [11]. In TMIH, dilated small bowel loops, directly abutting the anterior abdominal wall, lateral to the colon with convergence of engorged vessels at the entrance of the hernial orifice, and central displacements of the colonic segments are seen [4]. Intestinal volvulus, including midgut volvulus and closed-loop obstruction, is frequently mistaken for these hernias. During an exploratory laparotomy, a conclusive diagnosis is typically made [12]. Treatment of the asymptomatic TMIH is conservative, with operative intervention required for complicated types of TMIH. After midline laparotomy, thorough anatomical delineation is done, followed by reduction of herniated structures and closure of hernial defect. If there is perforation or necrosis of herniated bowel loops, resection and anastomosis should be done. There is little literature regarding the laparoscopic repair of TMIH. The laparoscopic approach helps during diagnostic dilemmas; however, there will be difficulty via laparoscopy in cases of bowel obstruction with distended bowel loops and necrotic or perforated bowels [12,13]. Most cases of TMIH reported in the literature have been managed with laparotomy, often due to the complexity of reducing bowel loops and addressing the defect in patients presenting with significant bowel distention and obstruction [6,8]. While laparoscopic approaches are generally preferred in minimally complicated cases, the feasibility of laparoscopic repair in TMIH remains debated. Laparoscopy is a promising approach in the diagnosis of IH, particularly in patients with unclear diagnoses or less severe obstruction. However, in more complicated cases involving extensive bowel distention or ischemia, as seen in our patient, laparotomy is still considered the gold standard due to the need for more extensive bowel inspection, adhesion release, and defect closure [12,13]. In our case, the patient underwent an exploratory laparotomy, which allowed for direct inspection and successful reduction of the hernia without bowel ischemia or perforation. A recent study on laparoscopic repair of TMIH highlighted some challenges with the laparoscopic approach, especially in cases of advanced bowel obstruction or necrosis, where the manipulation of severely distended bowel loops can be technically difficult [12]. In contrast, our case presented with non-perforated bowel obstruction and had a successful outcome following open surgery, underlining the importance of individualized surgical decision-making based on the patient's clinical status and the severity of the hernia. The current case adds new insights to the existing literature by highlighting the potential for TMIH in elderly patients, a demographic that has not been as widely discussed in previous reports. Most published cases focus on younger individuals without significant comorbidities, which may influence the choice of surgical approach [1,8,11,12]. In the differential diagnosis of intestinal obstruction due to internal hernia (IH), it is important to consider conditions such as torsion, primary volvulus, adhesions, and rare neoplastic conditions. Torsion and volvulus can present similarly to IH with bowel obstruction, though the characteristic “whirlpool sign” on CT imaging can help distinguish them. Adhesions, a more common cause of obstruction, should be considered, particularly in patients with prior abdominal surgeries. Rare neoplastic conditions like mesenteric tumors may also mimic the symptoms of IH. Early recognition of these differential diagnoses is critical to guide appropriate management and prevent complications [2,4].

## 4 Conclusion

Internal hernias (IH), particularly through congenital transverse mesocolic defects, are rare causes of intestinal obstruction, posing a diagnostic challenge. Timely diagnosis with imaging modalities like CECT and prompt surgical intervention are crucial for preventing bowel necrosis and reducing morbidity. This case underscores the necessity of considering congenital mesocolic defects in the differential diagnosis of unexplained intestinal obstruction, especially in elderly patients, where diagnostic challenges are greater. With appropriate surgical repair, the prognosis is favorable (as in our case), as demonstrated by the patient's successful recovery and lack of recurrence.

## Author contribution

1. Constructing hypothesis for the manuscript- Samrat Shrestha, Suresh Maharjan.
2. Planning methodology to reach the conclusion: Samrat Shrestha, Bijay Raj Bhatta, Suresh Maharjan.
3. Organizing and supervising the course of the article and taking responsibility: Samrat Shrestha.
4. Patient follow-up and reporting – Sabin K Ghimire, Mecklina Shrestha, Bijay Raj Bhatta.
5. Logical interpretation and presentation of the results- Samrat Shrestha, Suresh Maharjan, Mecklina Shrestha, Bijay Raj Bhatta, Sabin K Ghimire.
6. Construction of the whole or body of the manuscript- Samrat Shrestha, Bijay Raj Bhatta, Suresh Maharjan, Mecklina Shrestha, Sabin K Ghimire.
7. Reviewing the article before submission not only for spelling and grammar but also for its intellectual content- Samrat Shrestha, Suresh Maharjan, Mecklina Shrestha, Bijay Raj Bhatta, Sabin K Ghimire.

## Consent

Written informed consent was obtained from the patient for publication of this case report and accompanying images. A copy of the written consent is available for review by the Editor-in-Chief of this journal on request.

## Ethical approval

Ethical approval is waived at our institution (National Academy of Medical Science, Bir Hospital) and this study was exempt from ethical approval at our institution, as this paper reports a single case that emerged during a normal surgical case report.

## Guarantor

Samrat Shrestha.

## Research registration number

Not applicable.

## Funding

This research did not receive any specific grant from funding agencies in the public, commercial, or not-for-profit sectors.

## Conflict of interest statement

The authors have no conflict of interest to declare.
